# Health, dependency and caregiving: barriers to economic activity among individuals aged 50 to 69 years in Mexico

**DOI:** 10.1590/0102-311XEN120223

**Published:** 2024-07-29

**Authors:** Carlos Fernando Félix-Vega, Jeroen Spijker, Pilar Zueras

**Affiliations:** 1 Centre d’Estudis Demogràfics, Universitat Autònoma de Barcelona, Bellaterra, España.

**Keywords:** Disabled Persons, Health Services for Persons with Disabilities, Caregivers, Long-term Care, Personas con Discapacidad, Servicios de Salud para Personas con Discapacidad, Cuidadores, Cuidados a Largo Plazo, Pessoas com Deficiência, Serviços de Saúde para Pessoas com Deficiência, Cuidadores, Assistência de Longa Duração

## Abstract

In Mexico, the economically active population aged over 50 years has been increasing in recent years. Due to their age, these workers may experience health deterioration and require some form of care. However, only formal employment is associated with better access to health services and pensions. At the same time, these workers may also need to care for children, sick partners or dependent older adults, which limits their time available for employment. This study examined the association between disability, receiving and providing care and access to health services, and economic activity among adults aged 50 to 69 in Mexico in 2015 and 2018. Multilevel modeling was conducted using data from the *Mexican Health and Aging Study* (MHAS). The MHAS is a longitudinal panel study of adults aged 50 years and older. The study sample included data from 8,831 observations from 2015 and 10,445 observations from 2018. Those living with some degree of disability and receiving care were found to be less likely to be economically active than those living with disability and not receiving care. Similarly, individuals who care for someone were also found to be less likely to be employed. Furthermore, the data suggested that individuals without access to health services were more likely to be economically active. For individuals aged 50 to 69 years, health and care issues were factors that limited economic activity status. In family-oriented societies with weak welfare states, the right to health is partial for the population and care is traditionally the responsibility of women, which exacerbates gender inequalities and has a differential impact on paid work for men and women.

## Resumen

La población de México económicamente activa mayor de 50 años de edad se ha incrementado en los últimos años. Estos trabajadores en esta edad pueden sentir empeorar su salud y requerir algún tipo de atención. Sin embargo, solamente el empleo formal dio un mejor acceso a los servicios sanitarios y las pensiones. Al mismo tiempo, esta población también puede necesitar cuidar a niños, a la pareja enferma o a una persona mayor dependiente, lo que limita su tiempo disponible para trabajar. Este estudio evaluó la asociación entre la discapacidad, recibir y brindar asistencia y acceso a los servicios sanitarios, y la actividad económica de adultos de entre 50 y 69 años en México en el período de 2015 y 2018. Se trata de un enfoque de modelado multinivel que utiliza datos del *Estudio Nacional de Salud y Envejecimiento en México* (ENASEM). ENASEM es un estudio longitudinal con adultos de 50 años o más. La muestra del estudio estuvo conformada por 8.831 observaciones en 2015 y 10.445 en 2018. Las personas que viven con algún grado de discapacidad y reciben atención tienen menos probabilidades de ser económicamente activas en comparación con las que viven con discapacidades y no reciben atención. Del mismo modo, las personas que brindan atención también tienen menos probabilidades de tener un trabajo. Además, los datos destacan que las personas sin acceso a los servicios sanitarios tienen más probabilidades de ser económicamente activas. En las personas de entre 50 y 69 años, los problemas de salud y la atención son factores que restringen la condición de la actividad económica. En las sociedades orientadas a la familia con estados de bienestar débiles, el derecho a la salud es parcial a la población y el cuidado es tradicionalmente realizado por las mujeres, lo que empeora las desigualdades de género y tiene un impacto diferencial en el trabajo remunerado para hombres y mujeres.

## Resumo

No México, a população economicamente ativa com mais de 50 anos tem crescido nos últimos anos. Devido à idade, estes trabalhadores podem apresentar piora da saúde e necessitar de algum tipo de cuidado. No entanto, apenas o emprego formal está atrelado a um melhor acesso aos serviços de saúde e aposentadorias. Ao mesmo tempo, indivíduos dessa população também podem precisar cuidar de crianças, um companheiro doente ou idoso dependente, o que limita seu tempo disponível para o trabalho. Este estudo examinou a associação entre deficiência, receber e fornecer cuidados e acesso a serviços de saúde e a atividade econômica entre adultos de 50 a 69 anos no México em 2015 e 2018. Trata-se de uma abordagem com modelagem multinível utilizando dados do *Estudo Nacional sobre Saúde e Envelhecimento no México* (ENASEM)*.* O ENASEM é um estudo longitudinal painel com adultos com 50 anos ou mais. A amostra do estudo incluiu dados de 8.831 observações em 2015 e 10.445 em 2018. Aqueles que vivem com algum grau de incapacidade e recebem cuidados têm menor probabilidade de serem economicamente ativos em comparação com aqueles que vivem com incapacidades e não recebem cuidados. Da mesma forma, os indivíduos que prestam cuidados também são menos propensos a terem um emprego. Além disso, os dados sugerem que indivíduos sem acesso a serviços de saúde têm maior probabilidade de serem economicamente ativos. Em indivíduos na faixa etária de 50 a 69 anos, problemas de saúde e cuidados são fatores que restringem a condição de atividade econômica. Em sociedades orientadas para a família com estados de bem-estar social fracos, o direito à saúde é parcial para a população e o cuidado é tradicionalmente assumido pelas mulheres, o que agrava as desigualdades de gênero e tem um impacto diferencial no trabalho remunerado para homens e mulheres.

## Introduction

Mexico is facing an aging process similar to that of other middle-income countries, but characterized by a faster transition from a young to an aging population, as has been the case in high-income countries ^1^. While this demographic transition is well established in most developed countries, it is still a relatively new phenomenon in Latin America, the Caribbean, and other low- and middle-income regions. Nonetheless, it is expected to become the dominant demographic dynamic in future decades ^2^ ^,^ ^3^ ^,^ ^4^ ^,^ ^5^. According to World Health Organization parameters, Mexico is classified as having a moderately advanced degree of aging. This means that the country has a total fertility rate of less than 2.5 children per woman, and between 10% and 14% of its population is over 60 years old. This demographic situation is similar to that of 12 other Latin American countries, including Colombia, Peru, and Venezuela ^2^ ^,^ ^6^.

Mexico’s National Institute of Statistics and Geography (INEGI, acronym in Spanish) projects that the proportion of people aged over 60 in Mexico will increase from 12% in 2020 to 23% in 2050 ^7^. In addition, in 2020, 31% of households had at least one adult aged 60 years or older and 27% of households were headed by an adult aged 60 or older ^8^. Due to their age, older adults are likely to have specific needs associated with physical and/or mental health problems that may require personal care. At the same time, however, they may have few or no economic resources to facilitate their transition into economic inactivity. This may be due to a lack of pensions and/or access to public health care services, which force them to remain economically active ^9^. The reasons for older adults to remain economically active are therefore different from those of younger populations ^10^.

Data from the *Mexican Health and Aging Study* (MHAS) show that in 2012, 36% of the surveyed population aged 50 years or older were economically active. This proportion increased to 38% in 2015 and further to 42.5% in 2018 ^11^. Despite similarities with other Latin American countries ^10^ ^,^ ^12^ in terms of decreasing labor force participation with age, economic activity among older individuals in all age groups increased from 2012 to 2016 ^10^. However, economic activity among older adults is generally carried out in the informal sector. In 2018, 54.5% of those aged 50 to 54 worked in the informal sector, a figure that increased to 59% for those aged 55 to 59 and 72.5% for those aged 60 and over ^13^, and the number of older workers in informal jobs is expected to continue to increase ^14^ ^,^ ^15^ ^,^ ^16^. This trend is concerning, as informal jobs not only conditions and defines access to public health services due to the lack of health insurance coverage, but also precludes enrollment in social security programs and access to pensions. For this reason, the age range of 50 to 69 years was chosen for this study, even though the legal age for access to pensions in Mexico is 65 years. This is because the reality is that many workers with informal careers are forced to remain in the labor market even beyond the age of 65 ^17^. In terms of health, individuals aged 50 to 69 are considered a vulnerable population, as they are prone to health deterioration. For instance, in 2018, 10.6% of individuals in this age group were impaired to some degree in performing basic and instrumental activities of daily living ^11^. In addition, some members of this age group require care themselves, while others care for household members, including their own children, parents, or someone else who is ill or disabled ^18^. These circumstances may negatively affect the likelihood that Mexicans aged 50 to 69 will actively participate in the labor market. Even those who work may be dependent on others to carry out certain activities of daily living. The level of dependence is related to their physical and cognitive abilities ^19^. Previous literature has documented how health status and dependency can affect work activity in adults aged 50 and older; specifically, living with dependency has been associated with fewer years of paid work, a lower likelihood of men being economically active, and poor work performance due to physical limitations ^20^ ^,^ ^21^ ^,^ ^22^.

Regarding care, it is important to consider that individuals aged 50 to 69 may not only need care, but are often responsible for caring for children, sick individuals or older adults. This limits their time available for economic activities ^23^ ^,^ ^24^ ^,^ ^25^ ^,^ ^26^ ^,^ ^27^. Previous research suggests that people aged 40 and over who spend time caring for others tend to reduce their work hours or leave the labor market ^27^ ^,^ ^28^.

In sum, older individuals may require care due to their health conditions and disabilities while also being responsible for caring for others.

Adding to the complexity of explaining the labor force participation of individuals aged 50 to 69, Peláez & Ferre Lues ^29^ acknowledged the crucial role played by insufficient medical services during this stage of life as a significant determinant of economic activity. This is because formal employment often provides access to medical services and pensions. Consequently, a lack of formal employment during one’s economically productive years can have the opposite effect, as inadequate access to health services can lead to higher economic activity at older ages ^9^ ^,^ ^29^.

It is noteworthy that, since the late 1970s, the Mexican State has sought to create legislation and mechanisms to care for the older population in order to reduce economic hardship and limited access to healthcare and care provisions. Despite these developments, Mexico still has an insufficiently consolidated welfare system to ensure security for older people who should theoretically be retired or are in their final years of work ^9^ ^,^ ^30^. Accordingly, older workers, along with their nuclear families and social organizations, have taken on the responsibility, in the absence of the State, of securing economic resources and health care while providing care for the elderly ^25^ ^,^ ^26^ ^,^ ^31^ ^,^ ^32^ ^,^ ^33^.

This research contributes to the understanding of the need for pension programs to promote the well-being of older adults in Mexico. The Mexican State is currently responding to the diverse needs for support in old age via pension programs. The insights derived from the variables analyzed serve as a foundational reference for future evaluations of the policy implemented in 2019.

## Methods

### Data source

Data from the MHAS were used. The MHAS is a longitudinal panel study of adults aged 50 years and older in Mexico with national urban and rural representativeness. The study used a random, probabilistic, two-stage, stratified, and conglomerate sample. The design and objectives of the study were modeled based on the *Health and Retirement Study* (HRS) in the United States. The baseline survey was conducted in 2001 and included adults born in 1951 or earlier, with 15,186 respondents. Follow-up face-to-face interviews were conducted in 2003, 2012, 2015, 2018, and 2021. The MHAS provides information at the household and individual levels and has access-restricted data on community services and causes of death at the municipal and community levels. It collects information on health, income, socioeconomic conditions, family, economic activities, and time use. For more detailed information on the survey, see MHAS ^11^.

This study used data from the 2015 and 2018 survey rounds, excluding the 2021 sample to avoid potential bias in the results caused by the unique circumstances of the COVID-19 pandemic, which affected employment, health, and care arrangements. Respondents aged 50 to 69 years who participated in one or both waves and provided complete information on their economic activity were selected. A total of 128 cases were excluded due to missing information on educational attainment, resulting in a final sample of 18,565 measurement occasions (observations) from 12,761 individuals (Table 1). Of this group, 45.8% had observations from both rounds, 31.7% contributed only to the 2015 sample, and 22.8% contributed to the 2018 sample.

**Table 1:** Sample characteristics.

Characteristics	Measurement occasions		
	2015 (n = 8,718)	2018 (n = 9,847)	Total (N = 18,565)
	%	%	%
Economic activity status			
0 = not active	51.23	43.18	46.94
1 = active	48.77	56.82	53.06
0 = no disability	86.70	89.38	88.13
Disability gradient based on disability and reception of care			
1 = 1 ADL and/or any IADL without care	4.87	4.01	4.42
2 = 1 ADL and/or any IADL with care	4.21	2.84	3.48
3 = 2+ ADL without care	1.36	1.06	1.19
4 = 2+ ADL with care	2.86	2.71	2.78
Caregiving			
0 = no	54.81	57.09	56.02
1 = yes	45.19	42.91	43.98
Access to health services			
0 = yes	90.88	89.28	90.03
1 = no	9.12	10.72	9.97
Age (years)			
0 = 50 to 54	15.92	29.11	22.92
1 = 55 to 59	26.96	26.89	26.92
2 = 60 to 64	24.83	22.09	23.38
3 = 65 to 69	32.29	21.91	26.78
Sex			
0 = female	58.8	56.34	57.59
1 = male	41.20	43.66	42.41
Educational level			
0 = incomplete elementary school	37.03	28.34	32.42
1 = primary school completed	23.26	22.13	22.66
2 = secondary school	21.12	24.70	23.02
3 = high school or more	18.59	24.83	21.90
Marital status			
0 = married/stable union	74.06	75.18	74.66
1 = single/divorced/separated	15.31	15.48	15.39
2 = widowed	10.63	9.34	9.95

ADL: activity of daily living; IADL: instrumental activity of daily living.

Source: prepared by the authors with data from the *Mexican Health and Aging Study* ^11^.

### Outcome

The dependent variable was economic activity status, defined as an economically active or economically inactive population.

### Covariates

#### Disability gradient and reception of care

Two variables were combined. First, an indicator of level of disability was constructed based on respondents’ reports of difficulty in performing instrumental activities of daily living (IADLs) and basic activities of daily living (ADLs). IADLs and ADLs are frequently used in studies on disability because they are measures of the performance of essential activities for independent living in older age ^34^ ^,^ ^35^ ^,^ ^36^. The MHAS collected information on the following ADLs: walking at home; showering; eating meals; getting in or out of bed; and using the toilet. In addition, it collected information on the following IADLs: cooking; shopping; counting money; and taking medication. Specifically, the categories were based on the number of ADL and IADL limitations: no disability; 1 ADL and/or any IADL; and 2+ ADLs. The same categorization was previously used by Spijker et al. ^37^. Second, individuals who received assistance with any ADLs and/or IADLs in the past three months were identified. The effects of the disability gradient and of the “receiving care” variable on economic activity were then tested, both separately and by combining the variables into one indicator. The latter indicator distinguished between five statuses: no disability; 1 ADL and/or any IADL without care; 1 ADL and/or any IADL with care; 2+ ADLs without care; and 2+ ADLs with care. The results indicated that it was the interaction between these two variables that accounted for more variation in economic activity.

#### Caregiving

Caregiving, a binary variable, referred to spending time caring for a sick or disabled adult or a child under 12 years of age during the past year, outside regular work responsibilities.

#### Access to health services

Access to health services, a binary variable, was constructed based on a question about eligibility for medical care from the following health services in Mexico: social security; ISSSTE (state workers); popular security (*Seguro Popular*, medical service for the population without social security); Pemex (oil workers); Militia (army or marines); private insurance; and/or others.

#### Control variables

The following control variables were included, as they are associated with economic activity status: five age groups (50 to 54; 55 to 59; 60 to 64; 65 to 69) ^38^ ^,^ ^39^; four educational levels (incomplete elementary school; primary school completed; secondary school; high school or more) ^40^ ^,^ ^41^; and three categories of marital status (married/stable union; single/divorced/separated; widowed) ^42^. Marital status was divided into only three categories to increase the number of cases and because of the small variation in results for similar categories. Time was considered in the analysis to control for model variance due to the presence of respondents in 2015, 2018, or both waves.

### Statistical analysis

Given the nature of the data, multilevel logistic regression was used in the multivariate analysis. As the MHAS is a panel, information nested in two levels was used. Level 1 corresponded to individuals and their time-constant variables, and level 2 contained observations and their time-varying information. The results show regression coefficients and, for a clearer reading of the explanatory variables, predictive margins of being in the labor force accompanied by 95% confidence intervals (95%CI). Men and women were analyzed separately. Five models estimated the association between dependent and independent variables.

Table 1 shows the distribution of the variable categories used in the analysis for the 2015 and 2018 waves and the aggregate for all observations.

To assess the appropriateness of multilevel modeling, the intraclass correlation coefficient (ICC) was calculated on the null model, i.e. without explanatory or control variables. The ICC indicated that 76% of the individual-level variance in economic activity status was due to within-individual variation between the two periods, justifying the multilevel approach. The ICC dropped to 66% after including the explanatory and control variables.

The models were specified as follows: model 1 (M1) included sociodemographic control variables; model 2 (M2) combined the disability and care indicators; model 3 (M3) analyzed caregiving; model 4 (M4) tested the effect of access to health services; and model 5 (M5) included the survey year to find out whether, net of the other factors included, there was a change in the employment status of 50-69-year-olds between 2015 and 2018. All models were differentiated by sex, as it is widely documented that gender determines working conditions and access to the labor market ^41^ ^,^ ^43^.

### Ethical statements

Publicly available secondary data were used. The studies were conducted in accordance with local legislation and institutional requirements. Written informed consent for participation was not required from participants or their legal guardians/next of kin according to national legislation and institutional requirements.

## Results

Table 1 shows that 57.6% of all observations referred to women. The age structure of the two samples was similar (23% to 27% of observations when combining both samples), although the 2015 sample was slightly older (e.g. 15.9% of participants in 2015 were aged 50 to 54, compared to 29.1% of participants in 2018), likely due to refreshment sampling in 2018. Regarding education, 32.4% of respondents had not completed primary school, while other education categories had similar proportions overall (21.9%-23%), although the 2018 sample had a slightly higher proportion of individuals with higher education. Most respondents were married/stable union (74.7% of all observations), followed by single/divorced/separated (a combined 15.4%), and widowed (10%), with few differences between the two years. Over half of the participants were economically active (53.1% of observations), a proportion that increased in 2018. Regarding disability and reception of care, 88.1% of the observations indicated an absence of disability, 4.4% described participants who 1 ADL and/or any IADL without care, 3.5% described participants who had 1 ADL and/or any IADL with care, 1.2% indicated respondents who mentioned having 2+ ADLs without care, and 2.8% described participants who had 2+ ADLs with care. In 44% of the observations, respondents provided care, and 90% of these respondents reported having access to health services.

Regarding the multivariate analysis, only control variables were included in M1. The predictive margins showed the probability of being economically active after controlling for other variables at their mean value. Older men and women were both found to be less likely to participate in the labor market, but there was a significant gender difference between these groups. In the youngest age group, the probability of being economically active was close to 1 for men (Table 2, M1) and around 0.6 for women (Table 3, M1). Educational level and marital status also had different effects on labor market participation depending on gender. Education had a negative association with labor market participation for men and a positive association for women. Marital status also showed opposite associations: married men had the highest average margins of labor market participation (0.85) compared with men with other marital statuses (although the differences were not statistically significant), while married women had the lowest average margins of labor market participation (0.23). Single, divorced and separated women had the highest probability of being economically active (0.51), although this probability was still much lower than that of men of any marital status. For both men and women, these values did not change significantly after adding other variables.

**Table 2:** Multilevel logistic regression models. Coefficients and predictive margins * of labor market activity between 2015 and 2018 among men aged 50 to 69 in Mexico. Models 1, 2 and 3.

	M1			M2			M3		
	Coefficients	Margins	95%CI	Coefficients	Margins	95%CI	Coefficients	Margins	95%CI
Year									
2015	-	-	-	-	-	-	-	-	-
2018	-	-	-	-	-	-	-	-	-
Age (years)									
50 to 54	Reference	0.989	0.981-0.991	Reference	0.972	0.964-0.979	Reference	0.972	0.964-0.979
55 to 59	-1.018	0.963	0.952-0.975	-0.974	0.943	0.931-0.955	-0.977	0.942	0.930-0.954
60 to 64	-2.736	0.828	0.796-0.861	-2.636	0.814	0.785-0.844	-2.636	0.814	0.785-0.843
65 to 69	-3.830	0.622	0.581-0664	-3.693	0.635	0.598-0.673	-3.705	0.633	0.633-0.671
Educational level									
Incomplete elementary school	0.483	0.888	0.867-0908	0.534	0.883	0.865-0.900	0.510	0.881	0.863-0.898
Primary school completed	Reference	0.840	0.812-0867	Reference	0.836	0.812-0.860	Reference	0.836	0.812-0.860
Secondary school	-0.213	0.815	0.783-0847	-0.327	0.801	0.772-0.830	-0.319	0.802	0.773-0.831
High school or more	-0.185	0.818	0.790-0847	-0.371	0.796	0.770-0.822	-0.357	0.798	0.771-0.824
Marital status									
Marrried/Stable union	Reference	0.851	0.834-0.868	Reference	0.840	0.825-0.856	Reference	0.840	0.825-0.856
Single/Divorced/Separated	-0.251	0.823	0.783-0.862	-0.249	0.815	0.779-0.850	-0.272	0.812	0.776-0.848
Widowed	-0.080	0.842	0.787-0.897	-0.092	0.831	0.781-0.881	-0.097	0.830	0.781-0.880
Disability gradient and reception of care									
No disability	-	-	-	Reference	0.871	0.855-0.886	Reference	0.870	0.854-0.886
1 ADL and/or any IADL without care	-	-	-	-0.856	0.771	0.711-0.831	-0.8773	0.772	0.712-0.831
1 ADL and/or any IADL with care	-	-	-	-2.826	0.456	0.358-0.553	-0.2.846	0.453	0.355-0.550
2+ ADL without care	-	-	-	-1.495	0.676	0.539-0.814	-1.514	0.674	0.537-0.811
2+ ADL with care	-	-	-	-4.644	0.183	0.104-0.261	-4.636	0.181	0.104-0.258
Caregiving									
No	-	-	-	-	-	-	Reference	0.847	0.831-0.863
Yes	-	-	-	-	-	-	-0.330	0.814	0.792-0.836
Access to health services									
Yes	-	-	-	-	-	-	-	-	-
No	-	-	-	-	-	-	-	-	-
Constant	4.335			4.506			4.623		
Random effect parameters (level 2)	6.453 (5.180-8.039)			5.741 (4.539-7.262)			5.742 (4.536-4.268)		
Constant variance									
Observations	7,873			7,873			7,873		
Cases	5,665			5,665			5,665		
LR test vs. logistic model: χ^2^(2)/probability	380.13/0.000			319.15/0.000			316.25/0.000		
ICC	0.662			0.635			0.635		

95%CI: 95% confidence interval; ADL: activity of daily living; IADL: instrumental activity of daily living; ICC: intraclass correlation coefficient; LR: likelihood ratio; M1: model 1; M2: model 2; M3: model 3.

Source: prepared by the authors with data from the *Mexican Health and Aging Study* ^11^.

* The margins are average predicted probabilities. Margins report average values after regression and average probabilities after logistic regression.

**Table 3:** Multilevel logistic regression models. Coefficients and predictive margins * of labor market activity between 2015 and 2018 among women aged 50 to 69 in Mexico. Models 1, 2 and 3.

	M1			M2			M3		
	Coefficients	Margins	95%CI	Coefficients	Margins	95%CI	Coefficients	Margins	95%CI
Year									
2015	-	-	-	-	-	-	-	-	-
2018	-	-	-	-	-	-	-	-	-
Age (years)									
50 to 54	Reference	0.573	0.537-0.608	Reference	0.568	0.533-0.602	Reference	0.573	0.539-0.607
55 to 59	-1.014	0.356	0.326-0.387	-1.023	0.356	0.326-0.386	-1.035	0.361	0.331-0.391
60 to 64	-2.011	0.188	0.164-0.213	-1.992	0.194	0.171-0.218	-2.036	0.195	0.171-0.129
65 to 69	-2.967	0.089	0.073-0.105	-2.900	0.097	0.079-0.114	-2.971	0.096	0.079-0.113
Educational level									
Incomplete elementary school	-0.018	0.238	0.213-0.263	0.016	0.249	0.224-0.274	-0.009	0.248	0.223-0.273
style="text-indent:12px">Primary school completed	Reference	0.240	0.212-0.269	Reference	0.247	0.218-0.276	Reference	0.249	0.221-0.278
Secondary school	0.695	0.351	0.320-0.382	0.673	0.353	0.322-0.384	0.699	0.359	0.328-0.390
High school or more	0.991	0.404	0.366-0.441	0.910	0.394	0.358-0.430	0.901	0.394	0.358-0.430
Marital status									
Marrried/Stable union	Reference	0.230	0.213-0.247	Reference	0.234	0.217-0.251	Reference	0.236	0.219-0.252
Single/Divorced/Separated	1.676	0.512	0.476-0.548	1.699	0.517	0.481-0.552	1.693	0.515	0.480-0.550
Widowed	0.865	0.367	0.327-0.407	0.889	0.374	0.334-0.413	0.901	0.376	0.337-0.415
Disability gradient and reception of care									
No disability	-	-	-	Reference	0.319	0.303-0.335	Reference	0.321	0.305-0.337
1 ADL and/or any IADL without care	-	-	-	-0.220	0.285	0.233-0.337	-0.225	0.286	0.235-0.338
1 ADL and/or any IADL with care	-	-	-	-1.688	0.114	0.075-0.151	-1.688	0.116	0.078-0.154
2+ ADL without care	-	-	-	-0.237	0.283	0.187-0.379	-0.259	0.281	0.187-0.376
2+ ADL with care	-	-	-	-2.193	0.078	0.043-0.112	-2.244	0.077	0.043-0.110
Caregiving									
No	-	-	-	-	-	-	Reference	0.357	0.336-0.377
Yes	-	-	-	-	-	-	-0.614	0.264	0.247-0.281
Access to health services									
Yes	-	-	-	-	-	-	-	-	-
No	-	-	-	-	-	-	-	-	-
Constant	-0.406			-0.288			0.073		
Random effect parameters (level 2)	6.924 (5.947-8.061)			6.843 (5.866-7.982)			6.765 (5.794-7.899)		
Constant variance									
Observations	10,692			10,692			10,692		
Cases	7,096			7,096			7,096		
LR test vs. logistic model: χ^2^(2)/probability	798.37/0.000			778.38/0.000			763.92/0.000		
ICC	0.677			0.675			0.672		

95%CI: 95% confidence interval; ADL: activity of daily living; IADL: instrumental activity of daily living; ICC: intraclass correlation coefficient; LR: likelihood ratio; M1: model 1; M2: model 2; M3: model 3.

Source: prepared by the authors with data from the *Mexican Health and Aging Study* ^11^.

* The margins are average predicted probabilities. Margins report average values after regression and average probabilities after logistic regression.

M2 shows that, all else remaining constant, individuals with higher levels of disability, particularly those who had 2+ ADLs (versus those without care, with 1 ADL and/or any IADL) and also received care, were less likely to be economically active. This was true for both sexes. The average margin of men without dependency was 0.87, but the probability of being economically active decreased by 10 percentage points with each increasing degree of disability for men who did not receive care (confidence intervals overlapped between the two higher levels of dependency). However, among men who received care, the probability of being active in the labor market was almost half as high for those with 1 ADL and/or any IADL (0.46) as for those without care, and only 0.18 for those with 2+ ADLs (Table 2, M2). In contrast, among women who did not receive care, the probability of being active in the labor market did not differ significantly by degree of dependency, but among women who did receive care, this probability was reduced to 0.11 for those with 1 ADL and/or any IADL and to 0.08 for those with the highest level of dependency (Table 3, M2). For both sexes, these values remained constant in subsequent models.

Informal caregiving was associated with lower economic activity. This was particularly the case for women, as spending time caring for another person reduced their marginal probability of being economically active from 0.36 to 0.26 (Table 3, M3). The effects of caregiving were lower for men, with average margins decreasing by only 4 percentage points, which was not statistically significant (Table 2, M3).

Access to health services (M4) had less of an impact on the economic activity status of women than of men. Men aged 50 to 69 without access to health services had the highest average probability of being economically active (0.89), net of the variables considered in the analysis (Table 4, M4). In contrast, access to health services moderately affected women (the proportion of women working was 0.35, compared to 0.00 for women with access to health services), although the differences were not statistically significant at the 0.05 level (Table 5, M4).

**Table 4:** Multilevel logistic regression models. Coefficients and predictive margins * of labor market activity between 2015 and 2018 among men aged 50 to 69 in Mexico. Models 4 and 5.

	M4			M5		
	Coefficientes	Margins	95%CI	Coefficientes	Margins	95%CI
Year						
2015	-	-	-	Reference	0.830	0.813-0.848
2018	-	-	-	0.088	0.839	0.823-0.856
Age (years)						
50 to 54	Reference	0.970	0.962-0.978	Reference	0.970	0.962-0.978
55 to 59	-0.959	0.941	0.928-0.953	-0.948	0.940	0.928-0.953
60 to 64	-2.589	0.812	0.783-0.842	-2.574	0.812	0.783-0.841
65 to 69	-3.632	0.637	0.600-0.674	-3.608	0.638	0.602-0.675
Educational level						
Incomplete elementary school	0.476	0.877	0.859-0.895	0.482	0.877	0.859-0.895
Primary school completed	Reference	0.834	0.810-0.859	Reference	0.834	0.810-0.858
Secondary school	-0.307	0.802	0.773-0.831	-0.311	0.801	0.772-0.830
High school or more	-0.333	0.799	0.773-0.825	-0.335	0.798	0.772-0.824
Marital status						
Marrried/Stable union	Reference	0.839	0.824-0.855	Reference	0.839	0.823-0.854
Single/Divorced/Separated	-0.356	0.802	0.764-0.839	-0.353	0.801	0.764-0.839
Widowed	-0.098	0.829	0.779-0.879	-0.099	0.829	0.779-0.879
Disability gradient and reception of care						
No disability	Reference	0.868	0.852-0.884	Reference	0.868	0.852-0.883
1 ADL and/or any IADL without care	-0.826	0.772	0.713-0.831	-0.831	0.771	0.711-0.830
1 ADL and/or any IADL with care	-2.820	0.454	0.357-0.551	-2.807	0.456	0.359-0.552
2+ ADL without care	-1.511	0.671	0.535-0.807	-1.513	0.670	0.534-0.805
2+ ADL with care	-4.566	0.188	0.110-0.266	-0.456	0.188	0.110-0.266
Caregiving						
No	Reference	0.845	0.829-0.861	Reference	0.845	0.829-0.860
Yes	-0.314	0.813	0.791-0.835	-0.309	0.813	0.791-0.835
Access to health services						
Yes	Reference	0.829	0.813-0.845	Reference	0.829	0.813-0.844
No	0.660	0.887	0.863-0.912	0.657	0.887	0.862-0.911
Constant	4.491			4.425		
Random effect parameters (level 2)	5.586 (4.404-7.084)			5.549 (4.371-7.045)		
Constant variance						
Observations	7,873			7,873		
Cases	5,665			5,665		
LR test vs. logistic model: χ^2^(2)/probability	306.78/0.000			304.21/0.000		
ICC	0.629			0.627		

95%CI: 95% confidence interval; ADL: activity of daily living; IADL: instrumental activity of daily living; ICC: intraclass correlation coefficient; LR: likelihood ratio; M4: model 4; M5: model 5.

Source: prepared by the authors with data from the *Mexican Health and Aging Study* ^11^.

* The margins are average predicted probabilities. Margins report average values after regression and average probabilities after logistic regression.

**Table 5:** Multilevel logistic regression models. Coefficients and predictive margins * of labor market activity between 2015 and 2018 among women aged 50 to 69 in Mexico. Models 4 and 5.

	M4			M5		
	Coefficientes	Margins	95%CI	Coefficientes	Margins	95%CI
Year						
2015	-	-	-	Reference	0.293	0.276-0.311
2018	-	-	-	0.156	0.317	0.299-0.334
Age (years)						
50 to 54	Reference	0.571	0.537-0.605	Reference	0.571	0.537-0.605
55 to 59	-1.026	0.361	0.332-0.391	-1.019	0.362	0.333-0.392
60 to 64	-2.025	0.196	0.172-0.220	-2.024	0.196	0.172-0.220
65 to 69	-2.955	0.097	0.080-0.114	-2.965	0.096	0.079-0.113
Educational level						
Incomplete elementary school	-0.010	0.247	0.223-0.272	0.001	0.250	0.225-0.275
Primary school completed	Reference	0.249	0.221-0.277	Reference	0.250	0.222-0.278
Secondary school	0.706	0.360	0.329-0.390	0.698	0.359	0.328-0.389
High school or more	0.911	0.395	0.359-0.431	0.890	0.392	0.356-0.427
Marital status						
Marrried/Stable union	Reference	0.236	0.220-0.253	Reference	0.237	0.220-0.254
Single/Divorced/Separated	1.675	0.512	0.477-0.547	1.678	0.512	0.478-0.547
Widowed	0.897	0.376	0.337-0.415	0.890	0.375	0.336-0.414
Disability gradient and reception of care						
No disability	Reference	0.321	0.305-0.337	Reference	0.321	0.305-0.337
1 ADL and/or any IADL without care	-0.226	0.286	0.235-0.338	-0.208	0.289	0.238-0.341
1 ADL and/or any IADL with care	-1.675	0.117	0.079-0.155	-1.657	0.119	0.081-0.158
2+ ADL without care	-0.234	0.285	0.190-0.380	-0.217	0.288	0.193-0.382
2+ ADL with care	-2.234	0.077	0.043-0.111	-2.237	0.078	0.044-0.112
Caregiving						
No	Reference	0.356	0.336-0.377	Reference	0.356	0.336-0.376
Yes	-0.606	0.265	0.247-0.282	-0.599	0.266	0.248-0.283
Access to health services						
Yes	Reference	0.301	0.286-0.317	Reference	0.302	0.287-0.317
No	0.321	0.351	0.306-0.396	0.311	0.350	0.305-0.395
Constant	0.034			-0.049		
Random effect parameters (level 2)	6.742 (5.773-7.874)			6.733 (5.763-7.865)		
Constant variance						
Observations	10,692			10,692		
Cases	7,096			7,096		
LR test vs. logistic model: χ^2^(2)/probability	760.89/0.000			755.55/0.000		
ICC	0.672			0.671		

95%CI: 95% confidence interval; ADL: activity of daily living; IADL: instrumental activity of daily living; ICC: intraclass correlation coefficient; LR: likelihood ratio; M4: model 4; M5: model 5.

Source: prepared by the authors with data from the *Mexican Health and Aging Study* ^11^.

* The margins are average predicted probabilities. Margins report average values after regression and average probabilities after logistic regression.

The final model included the year of the observations (M5). After adjusting for all covariates, the overall probability of being economically active from the ages of 50 to 69 increased for both sexes from 2015 to 2018, although not significantly (Tables 4 and 5, M5).

## Discussion

Our study provides evidence on Mexican adults aged 50 to 69 and their relationship with dependency, caregivers, and economic activity. The results reveal that men and women with disabilities who received care were less likely to be economically active than men and women with disabilities who did not receive care. At the same time, informal caregivers were found to be less economically active, especially women. Regarding lack of access to health services, our research found that its impact on economic activity status was lower for women than for men, although the differences were not statistically significant.

In this paper, we used data from the 2015 and 2018 MHAS and applied a multilevel approach. Our results show that among older workers, when different demands for care and health deterioration occurred simultaneously, economic activity status was partly determined by the degree of disability - even if no care was being received -, by the time spent caring for others, and by lack of access to health services.

Although, as expected, a higher degree of disability in Mexico led to a lower likelihood of being economically active, we found that for the same level of disability, people who received care were even less likely to be economically active. This may be due to the need for personal (instrumental) assistance as a result of functional decline or health care needs due to illness or general health decline. However, we could not determine whether the difference in labor force participation between those within the same disability category who received care and those who did not was due to heterogeneity in the disability category, as we could not control for ADL/IADL severity.

Aligned with the fact that individuals aged 50 to 69 may have (grand)children and partners or parents in need of care, our findings revealed that caregiving reduced the likelihood of participating in the labor market, especially for women. In Mexico, as in many other societies, caregiving is a gendered role, with women being the primary caregivers for children, the sick, and other dependents. Women face time constraints that require them to negotiate the time available for other activities, including paid work ^24^ ^,^ ^25^ ^,^ ^26^ ^,^ ^44^.

The results also showed that individuals with access to health services were less likely to be economically active. In the Mexican context, this is perhaps to be expected, as access to health services is not guaranteed for those who are not formally employed or their dependents. Without social security coverage, these individuals must seek alternative resources to meet their health and care needs, which makes them vulnerable ^9^ ^,^ ^29^. Despite political efforts since the late 1970s to improve the health and economic resources of older people in Mexico ^9^, lack of access to health services remains an important factor associated with the likelihood of being economically active. The Mexican government is currently working to implement a new non-contributory pension system for all people aged 65 and over. Given the ongoing discussions about the national care system among social and political actors, this study is important because it sets a precedent for comprehensive thinking about a solution for caregiving.

The impact of all three variables differed significantly by sex. Caregiving had a greater effect on the labor market participation of women, while health and lack of access to health services were more determinant for men aged 50 to 64. It is important to highlight the high proportion of men who remained active in the labor market despite having difficulty performing ADLs or IADLs. This is likely due to Mexico’s weak welfare system, which relies heavily on family or friends for care, usually provided within the household ^25^ ^,^ ^26^ ^,^ ^31^ ^,^ ^32^ ^,^ ^33^ ^,^ ^45^. At the same time, the decrease in household size and the reconfiguration of the family over the past 50 years has resulted in fewer caregivers available to meet the needs of subjects who require care ^14^ ^,^ ^26^ ^,^ ^44^ ^,^ ^45^ ^,^ ^46^. For women, those who have fewer resources in terms of household income or lack access to health care services are at a disadvantage compared to those who do or live in wealthier households and can choose to pay caregivers. Moreover, having been out of the labor market (temporarily) is likely to become a disadvantage for women who wish to re-enter it, as better jobs will be more difficult to access, predisposing these women to informal economic activities and underemployment, which also limits their access to social security and medical services ^9^ ^,^ ^29^ ^,^ ^45^.

These results indicate that the lack of access to health services for oneself or one’s household unit puts additional pressure on women to care for a relative and on men to continue working even when their health deteriorates. Unfortunately, universal access to health services is not a guaranteed right in Mexico, as the State has failed to adequately manage society’s resources. This places economically vulnerable individuals in the dilemma of seeking resources by working even as their own health deteriorates ^45^ ^,^ ^47^.

Further research is needed to better understand the economic activity of people approaching retirement and the transition to inactivity. Two key areas for research are: (a) examining personal, social and welfare State mechanisms that facilitate a smooth transition from economically productive life to inactivity while ensuring economic security in later years; and (b) investigating ways to increase the formality of employment to improve the quality and conditions of work, such as employment security, wages, paid leave, and legal protection for workers. This would also provide greater economic security beyond productive age, as many pension plans are only offered via formal employment arrangements.

## Conclusion

The economic activity status of individuals aged 50 to 69 in Mexico is influenced by disability, caregiving, and access to health services, but men and women are affected differently. Men may be forced to remain in the labor market, sometimes with declining health, in order to secure the economic resources needed to meet basic and health needs, while women sometimes take on caregiving roles for other household members who, like them, may not have access to health services. Policies should therefore prioritize the expansion of health insurance coverage and the establishment of specialized health care centers for older adults to address physical and psychological health issues.
